# 

**Published:** 1894-12

**Authors:** 


### CONTENTS—NOVEMBER, 1894.—VOL. X., NO. 6.

ORIGINAL COMMUNICATIONS—
Causes and Treatment of Chronic Middle Ear Catarrh. R. E. Moss, M. D., San Antonio . . . . . 267
Spinal Concussion. James Johnston, M. D., Houston . . . . . 273
A Case of Epilepsy. Dr. Isadore Gluck, Houston . . . . . 278
Puerperal Eclampsia. L. W. Hollis, M. D., Abilene . . . . . 282
A Case of Strangulated Hernia. Dr. A. L. Fuller, Houston . . . . . 286
CURRENT MEDICAL LITERATURE—
Notes on Dermatology. Isadore Dyer, M. D., New Orleans . . . . . 288
ABSTRACTS AND SELECTIONS—
State Health Officer's Report . . . . . 291
SOCIETY NOTES—
Mississippi Valley Medical Association, Hot Springs Meeting . . . . 300
North Texas Medical Association . . . . . 307
EDITORIAL—
Reform in Asylum Management . . . . . 309
MEDICAL NEWS AND MISCELLANY—
Numerous Paragraphs . . . . . 312
BOOK NOTICES— . . . . . 319
PUBLISHERS' NOTES . . . . . 323

